# Limited effect of radial oxygen loss on ammonia oxidizers in *Typha angustifolia* root hairs

**DOI:** 10.1038/s41598-020-72653-9

**Published:** 2020-09-24

**Authors:** Elena Hernández-del Amo, Iva Dolinová, Gal la Ramis-Jorba, Frederic Gich, Lluís Bañeras

**Affiliations:** 1 Group of Molecular Microbial Ecology, Institute of Aquatic Ecology (IEA), University of Girona, Edifici Aulari Comú-LEAR, C/Maria Aurèlia Capmany, 40, 17003 Girona, Catalonia Spain; 2 Institute for Nanomaterials, Advanced Technologies and Innovation, Technical University of Liberec, Studentská 2, 461 17 Liberec, Czech Republic; 3 Centro Tecnológico Leitat, Innovació 2, 08225 Terrassa, Spain

**Keywords:** Microbial ecology, Environmental microbiology

## Abstract

The benefits of plant–microbe interactions have been exploited extensively for nutrient removal. Radial oxygen loss in aquatic macrophytes potentially promotes nitrification and accelerates nitrogen removal through coupled nitrification–denitrification process. Nitrification is likely the limiting activity for an effective nitrogen removal in wetlands. In this work, we have quantified the effect of radial oxygen losses in *Typha angustifolia* plants in environments of contrasting salinities, including a temporary lagoon, a constructed wetland, and a river estuary. In all sites, radial oxygen diffusion occurred mainly at a narrow band, from 1 to 5 cm from the root tip, and were almost absent at the tip and basal sections of the root (> 5 cm). Root sections with active oxygen diffusion tended to show higher bacterial and archaeal densities in the rhizoplane according to 16S rRNA gene abundance data, except at higher salinities. Archaeal *amoA* /bacterial *amoA* gene ratios were highly variable among sites. Archaeal nitrifiers were only favoured over bacteria on the root surface of *Typha* collected from the constructed wetland. Collectively, radial oxygen loss had little effect on the nitrifying microbial community at the smaller scale (differences according to root-section), and observed differences were more likely related to prevailing physicochemical conditions of the studied environments or to long-term effects of the root microenvironment (root vs sediment comparisons).

## Introduction

Wetlands, either naturally occurring or constructed, contribute to nutrient removal from water and act as biodiversity reservoirs and landscape building, especially in land-to-sea transition areas^1^. Wetlands are characterized by the presence of dense vegetation areas (macrophytes), which are fundamental for the ecosystem function^2^. In general, vegetated areas affect the nutrient dynamics in the wetland either indirectly, by slowing down the water flow rate and prolonging residence times for biological transformations to take place, or directly, by actively participating in nutrient assimilation and promoting microbial growth^3–5^. More specifically, macrophytes are thought to provide an additional oxygen source for microorganisms growing in the rhizoplane (area in close contact to the root surface) and the rhizosphere (sediment area loosely attached but influenced by the root), thus promoting aerobic microniches in an essentially anaerobic environment, such as the wetland sediment^6, 7^. Several studies have addressed the importance of vegetation on microbial community structure in constructed wetlands, and have shown the impact of vegetation on nutrient removal efficiencies^3, 8–10^. On the contrary, few attempts have been made to study the specific microbial community in direct contact with the root surface, and decipher how spatial variations in oxygen diffusion due to radial oxygen loss (ROL, defined as the oxygen transfer from root aerenchyma to the rhizoplane and rhizosphere) would affect the composition of microbial communities and enhance the selection of aerobic bacteria and archaea^11, 12^.

Coupled nitrification and denitrification are relevant for nitrogen removal in wetlands and may account for the 80% of total N reduction^13, 14^. Environmental factors, such as temperature and aeration, would significantly affect nitrogen removal^15, 16^. The oxidation of ammonia to nitrate (nitrification) is primarily an aerobic autotrophic process, whereas denitrification, the step-wise conversion of nitrate to nitrogen gas, is mainly favoured in anaerobic environments^17^. The two opposite conditions for nitrification and denitrification can co-occur at the root surface of emergent macrophytes, in which ROL can provide oxic microniches for nitrification in an anaerobic environment^10, 12, 18^. ROL depends largely on plant physiology^11, 19^, and redox potential of the overlying sediment^20^, but depending on the species can account the majority of oxygen (up to 90%) found in the rhizosphere^11, 21^. To what extent does ROL stimulate nitrification and aerobic decomposition of organic matter has not yet been studied in detail at small scales.

Ammonia oxidation is an interesting model process to understand the effect of oxygen diffusion on the microbial community composition and abundance in roots mainly for two reasons. First, ammonia oxidation is catalysed by the ammonia monooxygenase protein (AMO), which can be quantitatively traced by studying the gene coding for the alpha subunit (*amoA*) of the multi-enzymatic complex^22^. Second, a very limited set of phylogenetically constrained groups of ammonia-oxidizing bacteria (AOB) and archaea (AOA) are able to perform this reaction, thus facilitating their study based on common phylogenetic markers, such as 16S rRNA sequences. AOB are mainly distributed within the *Nitrosomonas*, *Nitrosospira* and *Nitrosococcus* genera^23, 24^. In addition, it has been demonstrated that some *Nitrospira* species are able to carry out a complete oxidation of ammonia to nitrate, a process that have been named as Comammox (complete ammonia oxidation)^25, 26^. AOA are included in the phylum *Thaumarchaeota*^27^. Previous works have shown that AOA, AOB, and Comammox bacteria coexist in many environments^28–30^. In the root surface (rhizoplane) and the rhizosphere, AOA, AOB and Comammox populations could be affected by plant functional traits, such as phytochemicals exuded by roots^31^. Furthermore, it is hypothesized that ROL could also determine niche differentiation between the three microbial groups. ROL differences are supposed to occur along the root longitudinal axis due to the presence of apoplastic barriers^32^, but a direct effect of ROL on the abundance and composition of ammonia oxidizers in the rhizoplane has not been clearly stated so far.

We aimed to quantify the effect of ROL on the spatial distribution of ammonia oxidizing archaea (AOA) and bacteria (AOB) on the root surface (rhizoplane) of the narrowleaf cattail (*Typha angustifolia*)*. T. angustifolia* is particularly abundant in wetlands of the Mediterranean area and has potential implications on the oxygenation of sediments^33^. Moreover, *T. angustifolia* is one of the most commonly used plant species in constructed wetlands (CW) in the area^10, 34^. We hypothesized that root surface areas with higher ROL should exert a short-term selective pressure for nitrifying organisms, potentially affecting the nitrification activity. With this aim, we analysed the microbial community structure of the rhizoplane of several root hairs and rhizosphere collected from a CW, and along a river estuary. Locations were chosen in order to test for generalized effects of physicochemical conditions, in particular the effect of conductivity and redox gradient, over the AOB/AOA relative composition.

## Results and discussion

### Physicochemical properties

In all sampled environments, *Typha angustifolia* was the main emergent macrophyte and formed almost monospecific communities covering large surface areas. Moreover, sampled environments were located in a relatively small geographical area, thus minimizing weather effects (temperature, average rain, and solar radiation). Nevertheless, physicochemical conditions of the chosen environments were essentially different in terms of nutrient concentrations, salinity and redox potential. Unfortunately, nutrient concentrations in water were not recorded at the moment of sampling. Nitrate and ammonia concentrations in the two studied systems are relatively variable (ranging from undetectable to above 10 mg/L for NO_3_^−^ and NH_4_^+^, NO_2_^−^ is not detected above 0.2 mg/L) and highly influenced by the performance of the WWTP in the Empuriabrava area, or nutrient discharges due to agricultural activities in the Baix Ter area^10, 35, 36^. Water temperatures were around 26 °C, being slightly lower in Bassa de les Tortugues and higher in the Daró river mouth (Table 1). When samples were grouped according to the location (i.e. FWS-CW and Baix Ter), no significant differences of temperature were observed (U Mann–Whitney test, p > 0.05). Sampled environments spanned along a salinity gradient, ranging from slightly saline (conductivity values of 11.95 mS/cm^2^, estimated salinity 6.84 ppt), such as Bassa de les Tortugues, to low salinity fresh water, such as Rec Coll (0.823 mS/cm^2^, salinity 0.40 ppt). Water in the Empuriabrava FWS-CW showed typical conductivity values for the system in summer^37^.

**Table 1:** Main water physicochemical parameters in the studied sites.

	Temperature (°C)	Conductivity (mS/cm^2^)	Salinity (ppt)	Redox (mV)	Oxygen (ppm)	pH
**Empuriabrava FWS-CW**						
Europa Lagoon	27.6	3.84	2.02	23.7	0.42	8.6
Treatment cell 1	26.0	3.22	1.68	− 92.7	0.37	8.95
Treatment cell 2	24.4	3.38	1.77	70.0	0.29	8.99
**Baix Ter**						
Bassa Tortugues	22.8	11.96	6.84	170.5	9.51	7.18
River Daró mouth	28.7	1.13	0.56	90.5	13.05	7.31
Rec Coll	28.0	0.82	0.40	123.0	12.37	7.52

Water oxygen concentration, pH and Redox values were significantly different between the two geographical locations (U Mann–Whitney test, p < 0.05). The samples from the Empuriabrava FWS-CW were characterized by lower oxygen concentration (0.36 ± 0.06 ppm), higher pH (8.85 ± 0.21), and variable Redox values (from − 92 to 70 mV). In contrast, samples from the Baix Ter exhibited a higher oxygen concentration (on average 11.64 ± 1.89 ppm O_2_), relatively lower pH (7.34 ± 0.17), and high positive Redox values (from 90 to 170 mV) (Table 1). Lower oxygen concentrations in the Empuriabrava FWS-CW can be due to the relatively lower oxygen concentration at the Empuriabrava WWTP effluent and to the fact that planted soils lay on a impermeabilized clay layer minimizing diffusion effects compared to natural systems in the area^38^.

### Radial oxygen loss

**Figure 1:**
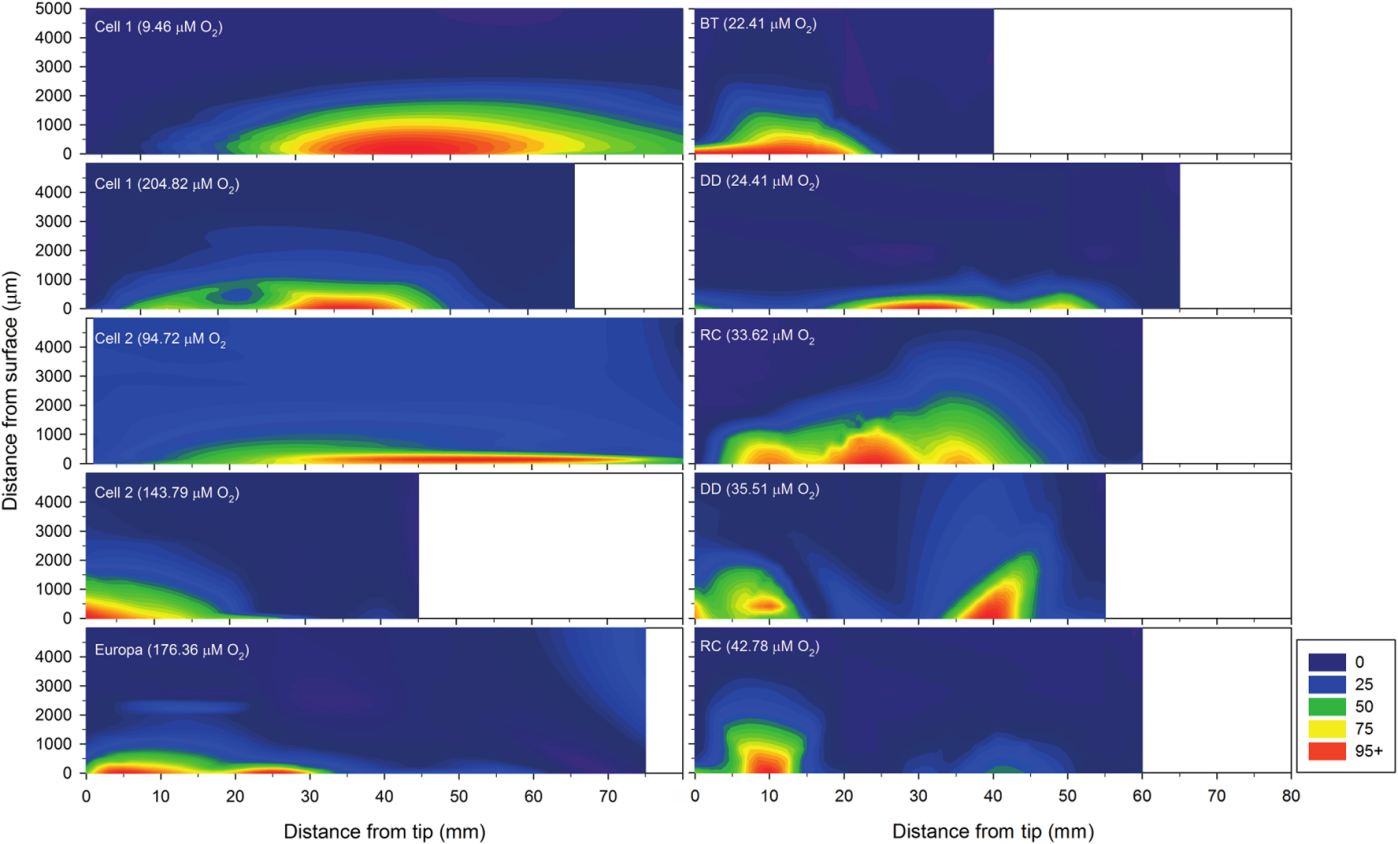
Contour plots showing the relative concentration of oxygen (% of maximum measured concentration per root, inset value in each graph) of 10 different roots at different points along the longitudinal axis. Left- Empuriabrava FWS-CW. Right- River Daró estuary. Cell 1 and 2- Sampling points 1 and 2 and the treatment cells of the Empuriabrava FWS-CW, *Europa* Europa Lagoon, *BT* Bassa de les Tortugues, *DD* Daró river mouth, *RC* Rec Coll.

Oxygen concentrations were measured orthogonally to the longitudinal axis of the root at different positions, from the tip to the base and were used to estimate potential radial oxygen loss (ROL). Ten different roots from *Typha angustifolia*, five from each of the two geographical sites, were analysed. In all cases, oxygen concentration increased at the root surface confirming diffusion from the root (Fig. 1). For most of the roots analysed, oxygen loss was higher in the middle section (from 1.5 to 4.5 cm from the tip) although a high variability of estimated diffusion rates was observed between roots, from 0.003 to 0.316 μmol O_2_/L/μm. Potential oxygen diffusion at the root tip ranged from non-detectable to values similar to those obtained in the middle section (from 3 × 10^–5^ to 0.196 μmol O_2_/L/μm). Basal portions (next to the plant junction) always showed the lower estimated diffusion values, ranging from 0.002 to 0.038 μmol O_2_/L/μm (Table 2). For the two geographical areas analysed, significant differences of potential oxygen diffusion rates were found between the middle section compared to either the tip or the basal sections (paired sample Wilcoxon test, p < 0.05), thus confirming that only a small section of *Typha angustifolia* roots is likely participating in the oxygenation of the sediment.

**Table 2:** Estimated oxygen diffusion rates and relative values at different root sections grouped according to the geographical area.

	Diffusion rate (nmol O_2_/L·μm)	Relative diffusion^a^	
**Empuriabrava FWS-CW**			
Root Tip (< 15 mm, n = 6)	12.5 ± 14.3	1	a
Middle Sect. (15 to 45 mm, n = 6)	51.5 ± 57.3	27.16 ± 49.17	b
Basal section (> 45 mm, n = 5)	8.4 ± 6.6	14.47 ± 22.93	ab
**Baix Ter**			
Root Tip (< 15 mm, n = 6)	10.4 ± 6.4	0.95 ± 0.12	AB
Middle Sect. (15 to 45 mm, n = 8)	16.5 ± 7.2	2.63 ± 2.05	B
Basal section (> 45 mm, n = 5)	7.4 ± 11.0	0.29 ± 0.17	A

Average values and SE are given. Statistically significant differences for homogeneous variance groups for each geographical area are indicated with letters in the rightmost column (Kruskal–Wallis test and Dunn’s test with Bonferroni correction, p < 0.05).

^a^Relativized to the potential diffusion rate measured at the root tip.

Plant aerenchyma is the main tissue by which oxygen can be exchanged between shoots and roots, ensuring an even distribution within plant tissues^39^. Aerenchyma is especially important in aquatic and wetland plants that grow in hypoxic sediments. Effective radial oxygen loss is proportional to the intensity of the concentration gradient at the root surface, the physical resistance of the dermis and cuticle to O_2_ diffusion in the radial direction, and the consumption of O_2_ by cells along this radial diffusion path^39, 40^. According to the equilibrium of production-consumption, actively growing root areas, such as the root tip, consume abundant oxygen and diffusion to the rhizosphere is diminished^6^. Similarly, older parts exhibiting a well formed and impermeable root barrier will also prevent oxygen diffusion into the adjacent sediment. Adventitious roots of many wetland species contain a barrier to ROL in the basal zones^12, 32, 41–43^. A barrier to ROL in basal root zones can enhance longitudinal O_2_ diffusion towards the apex, by diminishing losses to the rhizosphere^6^. However, diffusion from different portions of the root system can differ markedly but appears to be closely related to aerenchyma formation and maturation of the exodermal bands and suberin lamellae (ROL barriers). Particularly, *Typha* sp. has extensive casparian bands and suberin lamellae in all layers of the exodermis. Those differentiated cell structures are produced after extensive material deposition on the cell wall strengthening the tissue impermeability. Casparian bands and suberin provide resistance to gas exchange, minimizing losses in less active tissues^32^. As a result, O_2_ diffusion in *Typha angustifolia* roots is restricted to a narrow band (*circa* 4 cm in length) probably limiting the effect to the surrounding microbiota. To test this hypothesis, extensive microbiological analyses of the rhizoplane were performed using ammonia oxidizers as model organisms.

### Abundance of ammonia oxidizers

Bacterial cells were clearly visible in all sections of the root surface, albeit at relatively low densities (Supporting Figure S1). In order to determine changes on cell distribution along the root longitudinal axis, qPCR analyses of 16S rRNA and *amoA* genes for bacteria and archaea were used. Unfortunately, due to the low number of cells attached to the root surface, root sections from different root hairs had to be pooled in composite samples, precluding the use of paired-tests for this data set, which would have led to more conclusive results. Bacterial and Archaeal 16S rRNA genes ranged from 5.79 × 10^5^ to 1.09 × 10^9^ and 1.91 × 10^3^ to 2.49 × 10^8^ copies/g of fresh weight (FW) in roots, respectively (Supporting Figure S2). *amoA* genes were detected at much lower gene densities, and varied between 1.26 × 10^4^ and 8.06 × 10^5^ copies/g FW for AOB, and from 0.32 × 10^3^ to 4.03 × 10^5^ copies/g FW for AOA. Abundance of *amoA* genes, as well as for 16S rRNA bacterial an archaeal genes, was low in comparison to what have been previously found in *Typha* sp. colonizing environments of similar characteristics^44, 45^.

In order to determine differences in gene abundances between root sections, each sampling location was analysed separately. In the Empuriabrava FWS-CW, abundances of 16S rRNA *Bacteria* and *amoA Archaea* were significantly higher in the middle section compared to the root tip, and 16S rRNA *Bacteria* was significantly more abundant in middle compared to the basal section (Kruskal–Wallis and Dunn’s test with Bonferroni correction, p < 0.05). In the temporary lagoon, Bassa de les Tortugues, 16S rRNA *Bacteria* and *amoA Archaea* were found at significantly higher abundances in the middle than compared to the basal section of the root (Kruskal–Wallis and Dunn’s test with Bonferroni correction, p < 0.05) (Supporting Figure S2). In the Bassa de les Tortugues, a higher abundance of archaeal 16S rRNA and *amoA* genes was observed in the root tip section, despite no significant differences could be measured compared to the middle section of the roots. In agreement, two out of the ten intact roots studied to estimate ROL potential rates in laboratory conditions (one of them collected in the Bassa de les Tortugues, Fig. 1), exhibited a high diffusion rate at the very tip thus suggesting a potential effect on microbial abundance. However, a higher number of tips samples should have been analyzed in order to confirm these results. Moreover, in Daró River mouth, a higher abundance of *amoA Archaea* were found in the middle section compared to the basal section (Kruskal–Wallis and Dunn’s test with Bonferroni correction, p < 0.05). Finally, for Rec Coll samples, significantly higher abundances of all four genes were found in the middle section compared to the basal section. Generally speaking, the highest abundance of all genes in the rhizoplane was observed in the middle section, and the lowest in the basal section, although differences occurred between locations, suggesting that general physicochemical conditions of lagoons have an impact on the abundance of bacteria that remain attached to root at the low-scale level.

In previous works, higher abundances of *Bacteria* over *Archaea* were found in the *Typha* rhizosphere and rhizoplane thriving in similar environments as those analysed here^45, 46^. However, a direct analysis of ammonia oxidizers resulted in non-conclusive effects of plant roots on the relative abundance of archaea when compared to the surrounding sediment. For instance, archaeal *amoA* was more abundant compared to the bacterial counterpart in the rhizosphere of emergent aquatic macrophytes^46, 47^, agricultural crops, such as *Ipomoea batatas* and *Zea mays*^48^, and grassland vegetation^49^. In contrast, Sterngren et al. showed a higher nitrification activity of AOB, and Huang et al. found higher AOB densities compared to AOA in sediments and rhizospheres from *Ceratophyllum demersum* and *Potamogeton malainus* in Lake Taihu.

**Figure 2:**
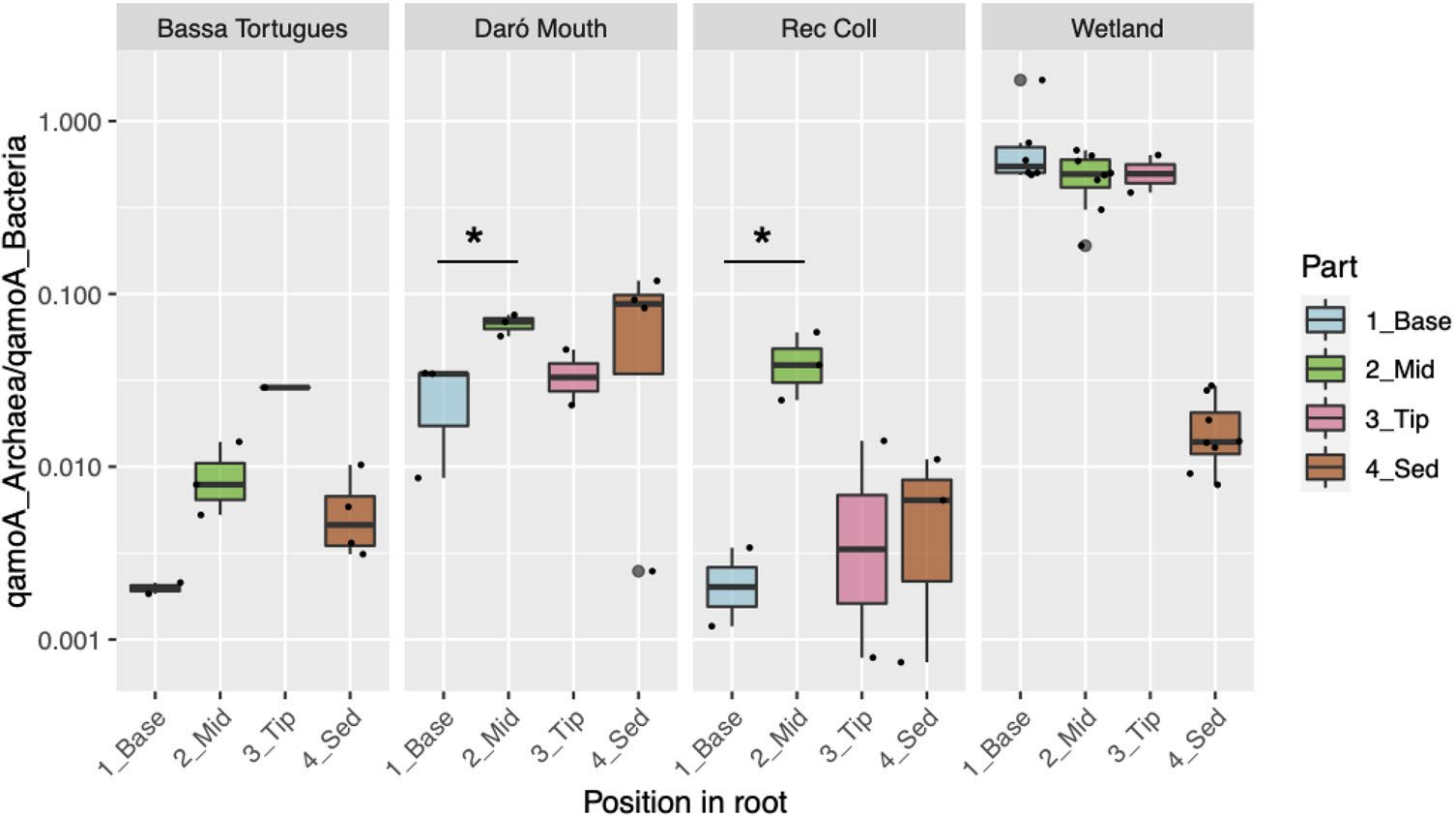
Box plot showing the ratio between ammonia oxidizing archaea and bacteria (q*amoA* Archaea/q*amoA* Bacteria) in the four studied environments according to the root section. Ratios in sediment samples are included for comparison. Distances from the root apex: Tips 0 to 15 mm, Middle 15 to 45 mm, Base- > 45 mm.

We checked for a selective effect of oxygen diffusion on either archaeal or bacterial ammonia oxidizers by comparing the abundance of *amoA Archaea*/abundance of *amoA Bacteria* (AOA/AOB) ratio of different root sections with the surrounding sediment (Fig. 2). Taking all data collectively, higher values of AOA/AOB were found in roots compared to adjacent sediments (Mann–Whitney test, p < 0.05; ranging from 0.001 to 0.747 and 0.001 to 0.119 for roots and sediments, respectively), being the differences more pronounced in the Empuriabrava FWS-CW and the Rec Coll, two environments of contrasting salinity and redox potential. Previous works have highlighted the importance of pH in the dominance of AOB over AOA in alkaline media, which holds also true for coastal lagoons^46^. Despite having relatively high pH values, we observed a higher AOA/AOB ratio in the Empuriabrava FWS-CW, in disagreement with the previous statement. Probably, sediment had different physicochemical variables compared to water, which could affect microbial communities in different way than roots, favouring AOB over AOA in sediment. AOA were described to be more sensible to salinity and to pH than AOB in microcosms and estuarine sediments^31, 50^; though it depends on the ecotype, archaea are able to survive in a wider range of salinity and oxygen concentration^51^. Considering the variability of gene abundances between samples of the same location, more analyses should be done to define a clear trend in this sense. In relation to changes according to root sections, significant differences of the AOA/AOB ratio were only found in Rec Coll and the Daró River mouth, in which archaea were significantly enriched in the middle compared to the basal section (Kruskal–Wallis and Dunn’s test with Bonferroni correction, p < 0.05). Rec Coll and Daró River mouth were the two environments showing the lowest water conductivity and no drastic seasonal changes in water flow are expected in the area. Responses of AOA/AOB ratios to various environmental factors have highlighted the importance of temperature, ammonia and oxygen level in addition to salinity and pH. AOA were suspected to be more sensitive to salinity and pH fluctuations than AOB according to the results of microcosms incubations and analyses of estuarine sediments^31, 50^. Although AOA are generally considered to have higher affinities for oxygen compared to AOB^51, 52^, some exceptions to this rule exist. For instance, Santoro et al. retrieved almost constant archaeal *amoA* gene copies in aerobic subterranean aquifer sediments with pore water at dissolved oxygen levels of 0.1–0.2 mM. Qin et al. showed that not all strains and isolates respond uniformly to increased oxygen concentration and some of them were stimulated at higher O_2_ concentrations. Könneke et al. reported the fully aerobic growth of *Nitrosopumilus maritimus* during cultivation and near-stoichiometric conversion of ammonium to nitrite, showing a limited effect of O_2_ concentration.

### Microbial community structure

Microbial communities at different root sections were studied on the basis of the 16S rRNA gene sequence. A total of 3,836,951 sequences passed quality filtering. On average, 66,154 sequences were obtained per sample (ranging from 23,001 to 142,783). Four samples (i.e. two replicates of the middle section, Cell 2 and Europa Lagoon, one replicate from the basal section, Bassa de les Tortugues, and one from sediment, Rec Coll) were discarded from the analysis due to poor sequencing depth. For comparisons between samples, a subset of 22,500 sequences per sample was randomly selected and used for alpha and beta diversity analyses. Observed richness (S_obs_, number of OTUs) varied between 158 and 2,916 (Supporting Table S1). Shannon’s and Phylodiversity diversity indices varied from 3.65 to 6.54 and from 10.85 to 100.33, respectively. Despite this variation, no significant differences were found across root sections and not between roots and sediment in alpha diversity indices of the analysed sites (Kruskal–Wallis and Dunn’s test with Bonferroni correction, p > 0.05).

The phylum *Proteobacteria* was the most abundant in all samples and accounted for more than 50% of all sequences (Supporting Figure S3). Results agree with what has been previously found in natural systems and in wastewater treatment plants^53, 54^. *Bacteroidetes*, *Chloroflexi* (mainly in CW) and *Firmicutes* were also abundant. Bacteria were dominant over Archaea in all samples, although differences did not match completely qPCR results. Archaea accounted from less than 0.01% of the total sequences reads in the sediment of the Daró River Mouth to up to 8% of sequences in the sediment of the Empuriabrava CW. According to qPCR, archaea to bacteria 16S gene ratio was significantly higher in the Empuriabrava sediment and rhizoplane in agreement with sequencing data. However, this higher proportion of archaea could not be solely attributable to a higher abundance of AOA. Only for the sake of comparison, AOA/16S rRNA archaeal genes was 1.9 × 10^–3^ (average value) in the Empuriabrava samples, and up to 1.4 × 10^–1^ for Rec Coll.

**Figure 3:**
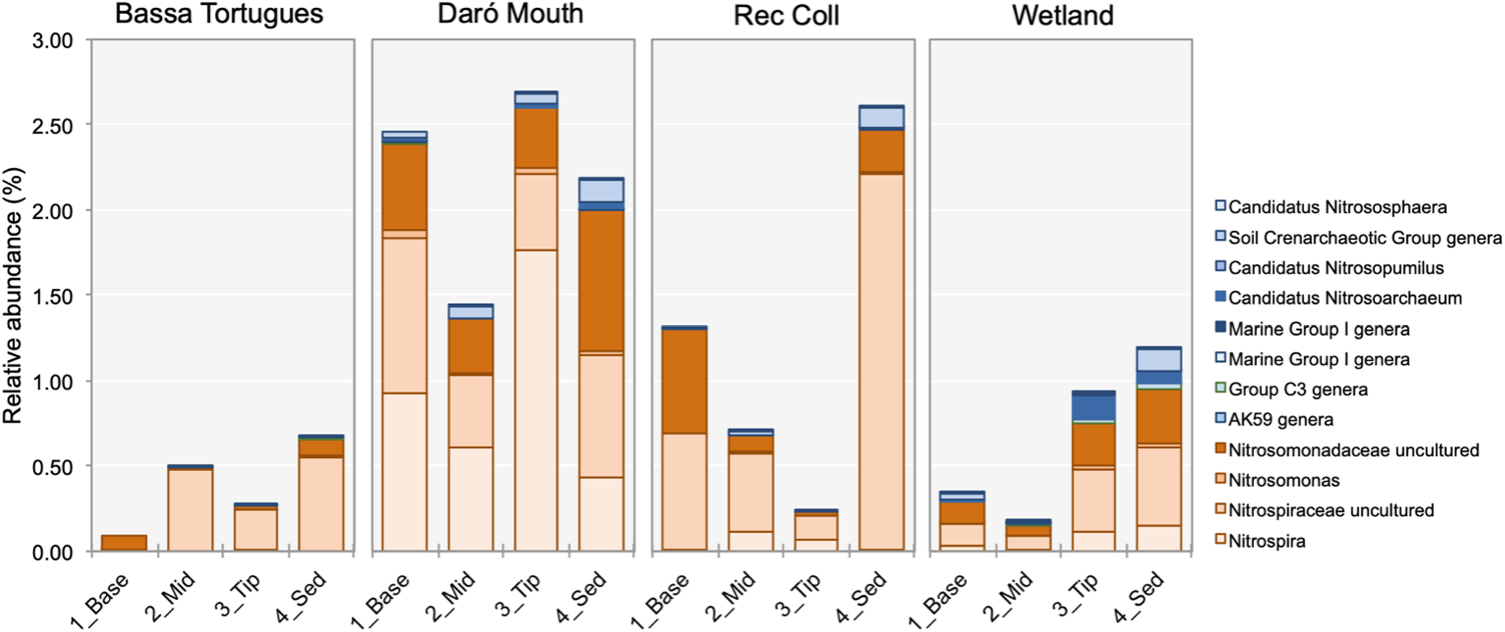
Relative abundance (per total number of sequences in samples) of identified ammonia oxidizers (groups as in Supporting Table S2). Blue colours—Ammonia Oxidizing Archaea. Orange colours—Ammonia Oxidizing Bacteria.

In order to specifically analyse changes in the composition of nitrifiers, sequences were classified down to the genus level and putative ammonia oxidizers were selected and analysed separately (Supporting Table S2). AOB were generally more represented than AOA, according to the relative abundance of sequences, which was in concordance to qPCR results (Fig. 3). Indicator species analyses did not result in conclusive results at the large-scale level (root *vs* sediment samples). Nevertheless, “*Candidatus* Nitrososphaera”, *Thaumarchaeota* Group C3, and “*Candidatus* Nitrosoarchaeum” and *Thaumarchaeota* Marine Group I were more prevalent in the sediment samples of the Empuriabrava FWS-CW, Bassa de les Tortugues, and in the Daró River Mouth, respectively. Those differences in potential indicator species among systems revealed a clear effect of the prevailing physicochemical conditions on the nitrifier community. In contrast, unclassified *Nitrosomonadaceae* and *Nitrospira* spp. were found to be related to root samples in the Daró River Mouth and Rec Coll, environments with the lowest salinity. *Nitrospira* spp. were detected in all samples (0.1 to 2.7% of sequences) being more abundant in the Daró River Mouth. Comammox *Nitrospira* spp. is supposed to be widely distributed in environments of diverging characteristics^24^, suggesting the possibility of the presence of microorganisms harbouring a complete nitrification gene set, especially in the Daró River Mouth.

Dissolved oxygen potentially has an advantageous effect on ammonia oxidizers. Due to differences in the affinity of nitrifying microbes for oxygen (AOA > AOB > NOB, including *Nitrospira*)^55^, environment selection on different populations was expected. High oxygen affinity makes AOA more competitive than AOB in hypoxic environments^51^. In fact, *Nitrosomonadaceae* and *Nitrospiraceae*, which had a lower affinity for oxygen, were the most represented taxa in the Daró River Mouth and Rec Coll, where the highest oxygen concentrations were found, especially in root samples. Common estuarine AOB, such as *Nitrosospira* spp., could not be unequivocally determined at the genus level in the samples analysed, although the proportion of unassigned *Nitrosomonadaceae* sequences was relatively high within nitrifiers’ related sequences. According to sequences assigned to genera, *Nitrosomonas* spp. were dominant on those systems. *Nitrosomonas* is commonly found in freshwater environments with relatively high C/N ratios or low total N conditions^24, 56^, conditions similar to those described for the studied environments.

### Root vs sediment differences in nitrifiers community structure

Differences in the structure of microbial communities were analysed with a Principal Coordinates Analysis (PCoA), based on the Unifrac weighted distance matrix. Two clusters were observed in PCoA samples distribution according to the two studied geographical sites (Supporting Figure S4), which was confirmed by PERMANOVA (pseudo-F = 10.716, p = 0.001). Redox and pH were correlated to the distribution of samples (Spearman’s test, r^2^ > 0.8). pH and Redox potential has been described as having an opposite effect on the structure of sediment microbial communities of constructed and natural wetlands^57–59^.

**Figure 4:**
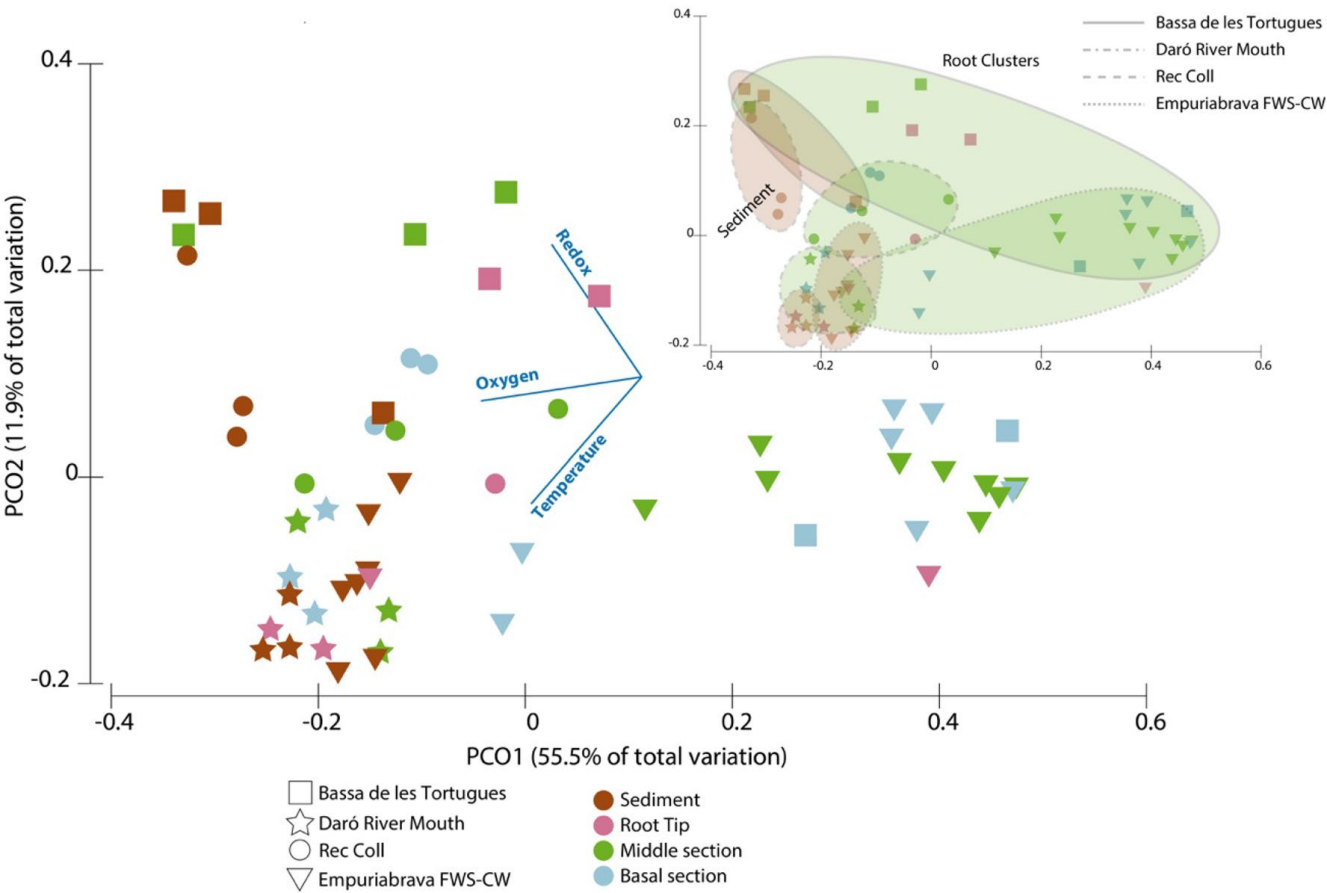
PCoA distribution of samples according to putative ammonia oxidizers community composition determined by Unifrac weighted matrix. Vectors show the correlation of environmental variables to PCoA axis 1 and 2. Sample labelling, Cell 1 and 2- Sampling points 1 and 2 and the Treatment cells of the Empuriabrava FWS-CW, *EE* Europa Lagoon at the Empuriabrava FWS-CW, *BT* Bassa de les Tortugues, *DD* Daró river mouth, *RC* Rec Coll. Inset showing clusters for root (green) and sediment (brown) samples in the four sampled environments.

In order to infer changes in the ammonia oxidizing community in the sampled sites, ordination plots (PCoA based on Unifrac weighted distances) were constructed considering only those sequences assigned to any of the taxa depicted in Fig. 3. Similar to what has been observed for the analysis of the total microbial community, samples grouped according to the geographical area (Fig. 4). Two groups could be clearly separated, the Baix Ter, and the Empuriabrava FWS-CW areas at a significant level (PERMANOVA test, pseudo-F = 15.727, p = 0.001). As a whole, samples distribution appeared to be correlated to redox potential, oxygen and temperature (Spearman’s test, r^2^ > 0.6). These results suggested an important effect of the environmental physicochemical properties in shaping the ammonia oxidizers community. Water oxygenation has been shown earlier to have a direct advantageous impact on the ammonia oxidizing community^45^, and differences in the affinity of nitrifying microbes for oxygen have been confirmed^55^, thus having a direct effect on the community composition. However, no clear effects of redox potential on ammonia oxidizers have been described, though an increase of redox potential (e.g. by oxygen release from the root or by low pH environment) could stimulate ammonia oxidation^24, 60^.

For all sampled sites, communities in the rhizoplane appeared to be different compared to the surrounding sediment, although significant differences were only found for the Empuriabrava FWS-CW, probably due to higher number of samples analysed. More specifically, middle and basal root sections appeared to have distinct ammonia oxidizing communities compared to sediments (PERMANOVA pseudo-F = 3.44, p < 0.01), whereas the two root tip samples that could be correctly analysed appeared at a long distance from each other. Despite the low number of samples, a similar distribution was observed in all sampling sites, that is basal and middle root sections harbouring a distinct nitrifier community. We can speculate on a progressive succession of nitrifier phylotypes eventually coming in contact with the root system at sections were radial oxygen loss occurs (middle section and tips). As root growth progresses, previously colonized areas develop stronger barriers to diffusion, thus slowing down the selective pressure of oxygen on the root surface (basal sections). At this point, little changes in relation to oxygen diffusion are supposed to occur in close contact to the root and nitrifying communities are potentially receiving a higher influence from the surrounding sediment. In this sense, the presence of microhabitats with evolving properties, sediment or different parts on the root, may have an impact on the community composition^5, 61^. However, to what extent is this occurring at the root surface during development should be further investigated using molecular proxies for activity of ammonia oxidizers.

## Concluding remarks

Our results suggested a limited effect of *Typha angustifolia* radial oxygen loss (ROL) on abundance and composition of ammonia oxidizers. Experiments were conducted considering only the rizhoplane, i.e. the sediment area in closest contact to the root, in order to avoid interferences with the adjacent sediment. At the large-scale (environment) plant effects were confounding and ammonia oxidising communities seemed to respond to physicochemical changes, i.e. pH, water oxygen concentration, and redox values. At smaller scales (intra-environment root vs sediment effects), roots seemed to have an effect on microbial communities, but those were not consistent in all sampled sites. For instance, despite the fact that AOB were always more abundant than AOA, roots seemed to exert a positive selection towards AOA only the rhizoplane only in the Empuriabrava FWS-CW. The lack of differences in the AOA/AOB ratio in the root sections prevented us to confirm the observed changes were due to an increased ROL. Despite the large variability observed between locations and roots, younger parts of the root (tips and middle sections) appeared to exhibit higher bacterial and archaeal gene abundances. Invariably, the lowest densities were found for the basal, less active, part of the root, thus confirming a tight plant-microorganism relationship, although those differences cannot be exclusively attributed oxygen leakage and other effects, such as organic matter exudation, need to be considered^12^. Specifically for nitrifiers, roots collected in environments with the lowest salinity (Daró River and Rec Coll) had a significant effect on the selection of AOA over AOB, and higher relative abundances of archaeal *amoA* occurred at positions were ROL was present. Despite being one of the most claimed effects of wetland vegetation, oxygenation at the root surface of *Typha angustifolia* seemed to have a rather limiting effect on abundance and diversity of microorganisms active responsible of an aerobic model process, such as nitrification. Nevertheless, experiments here were limited to studying changes in the abundance and composition of microbial communities based on DNA extractions, which may not be the most suitable for detecting short term changes. Further research using RNA-based methods or direct activity measurements (isotopic methods) would be needed to confirm these results.

## Materials and methods

### Study site and physicochemical conditions

**Figure 5:**
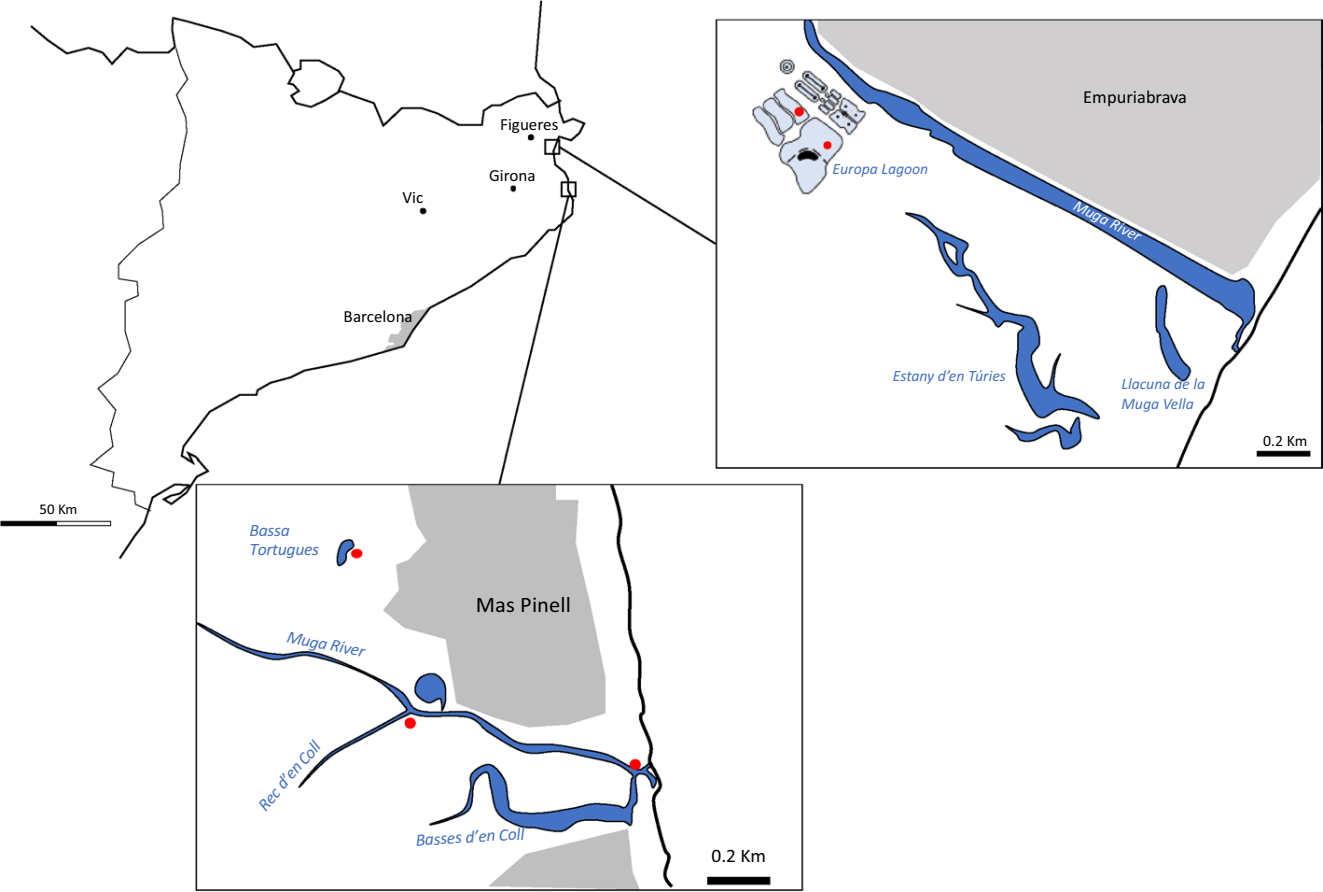
Location of sampling sites (red dots). Grey: urban areas. Line drawings were done from high quality ortophotographs of the areas obtained from the Institut Català de Cartografia (www.icc.cat/vissir3) using Adobe Ilustrator v17.0.

Narrowleaf cattail (*Typha angustifolia*) roots were collected from two different locations in the Mediterranean coast. In the naturally preserved area of the Parc Natural del Montgrí, Illes Medes i Baix Ter (42°00′33.3ʺ N 3°11′09.8ʺ E), three sampling positions were selected following a salinity and a redox gradient; the Daró River mouth (DM), at the site an irrigation channel discharges to the river (Rec Coll, 300 m up the river mouth, RC), and at an intermittent lagoon located in the northern side of the river bed (Bassa de les Tortugues, BT) (Fig. 5). The second sampling area was located in the Empuriabrava free water surface constructed wetland (FWS-CW, 42°14′40.4ʺ N 3°06′15.1ʺ E). The system was set in operation in May 1998, and functions as a reception system for the secondary effluent coming from the adjacent Empuriabrava WWTP. The plant is dimensioned for 70,000 equivalent inhabitants and basically composed of Biological + Nitrate treatment. The aim of the CW is to provide a final polishing of partially treated water, and contribute to additional removal of nutrients (nitrogen and phosphorus). Treated water is used for irrigation of gardening areas and a pitch & putt club, and to flood certain areas in the natural preserved area of Els Aiguamolls de l’Empordà (https://aiguamollsdelemporda.cat/) to prevent summer drought^62^. Samples were collected from treatment cells, and from the final water receiving Europa Lagoon (Fig. 5). In all sampling plots, narrowleaf cattail (*Typha angustifolia*) was the dominant macrophyte with densities above 5 individuals per square metre. Sediment and root samples were collected during July 2014 (FWS-CWs), and July 2015 (Baix Ter).

Physical and chemical monitoring of water was done at the different sampling sites. Temperature, conductivity, oxygen and pH were measured with a portable multiparametric probe (Yellow Spring Instruments 650MDS). Water samples (20 mL) were collected and analysed using ion chromatography (IC) for nitrate, nitrite and ammonium concentration as described previously^10^.

### Root and sediment sampling

In each location, three plant shoots were selected randomly in one square meter area. Plants were manually harvested taking special care in maintaining the roots intact. Immediately after collection, roots were thoroughly rinsed with water from the same sampling site to remove all loosely attached sediment^46^. Non-senescent and integer roots in which tips had not been damaged, were cut using sterile scissors, and rinsed twice in sterile isotonic solution. Recovered intact roots (> 6 cm in length) were separated and chilled to 4 °C in a portable ice box. Measurements of oxygen diffusion were performed within 4 h after collection, see below.

For molecular analyses, roots sections along the root longitudinal axis were obtained. Root tips were defined from 0 to 1.5 cm from the tip. Middle root section was defined from 1.5 to 4.5 cm. Basal root sections included all segments collected at distances higher than 4.5 cm from the root tip. These sections were separated according to the observed oxygen diffusion made in preliminary studies. Root sections were distributed in sterile plastic bags, chilled to 4 °C for transportation, and finally stored at − 20 °C. Due to the difficulty in sampling, only eight samples of intact tips could be processed satisfactorily, two from FWS-CW, two from Daró River mouth, two from Bassa de les Tortugues, and two from Rec Coll.

Additionally, three sediment cores were obtained from each sampling site. Sediment cores were obtained with a manual methacrylate core sampler (5 cm inner diameter). Vegetal debris and roots visible at naked eye were removed and the upper part of the core (0 to 4 cm depth) was selected^36^. Sediment samples were homogenized and stored at 4 °C for transportation. Once in the laboratory, sediments were distributed in 2 g aliquots, and frozen to − 20 °C.

### Scanning electron microscopy

For microscopy observations, root samples were fixed with 2.5% wt/vol glutaraldehyde in 0.1 M cacodylate buffer, pH 7.4, washed and dehydrated successively in ethanol. Finally, roots were dried at the critical point, and carbon evaporated. Examinations were performed in a scanning electron microscope FE-SEM S-4100 (Hitachi, Tokyo, Japan) at the Serveis Tècnics de Recerca (STR, Universitat de Girona). Digital images were collected and processed using the Quartz PCI measurement software (Quartz Imaging Corporation, Vancouver, Canada)^63^.

### Estimation of radial oxygen diffusion

**Figure 6:**
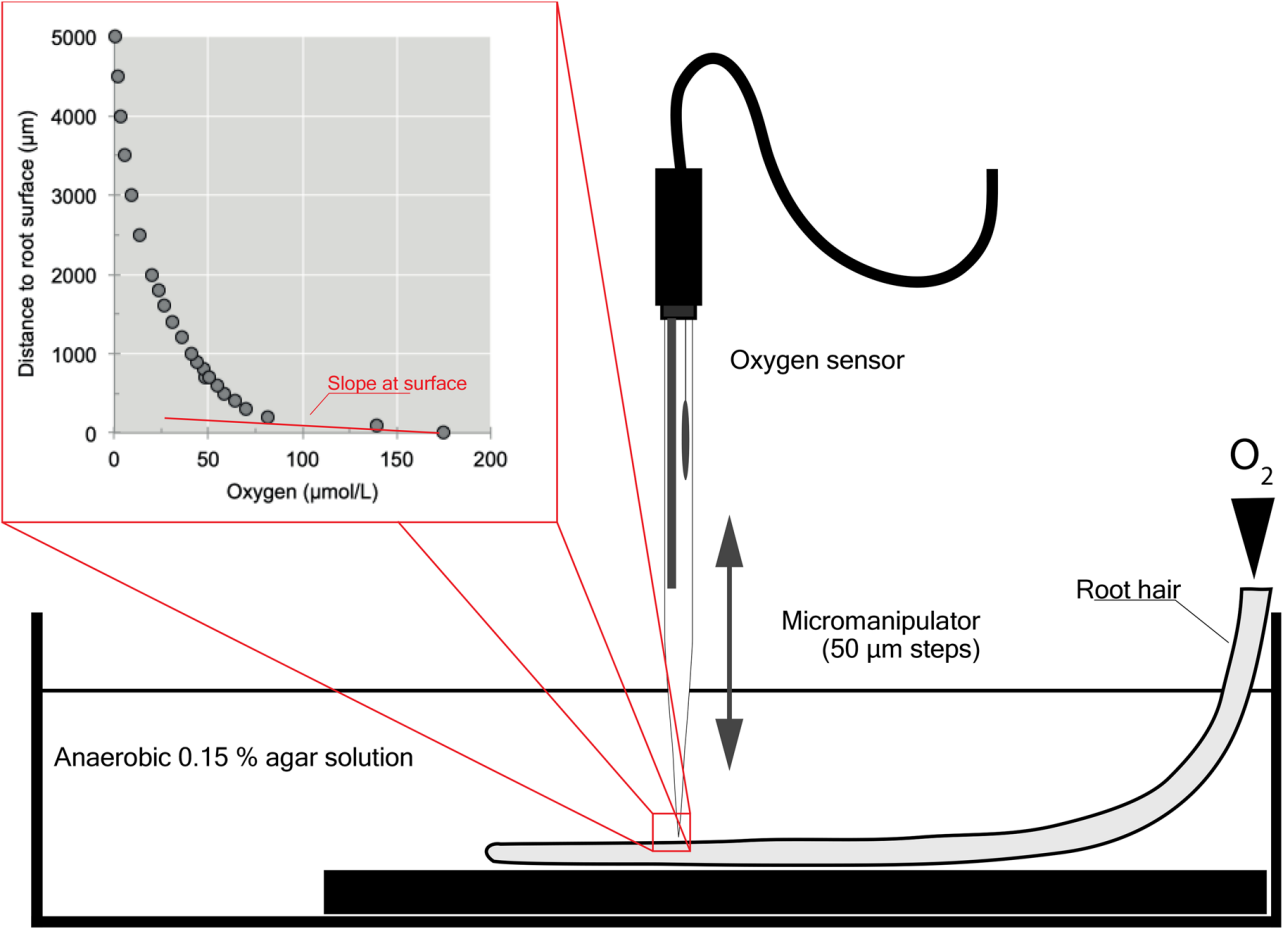
Diagram of the set-up used to monitor oxygen profiles at different positions along the root surface. Micromanipulator was moved in the *xy* axis to locate the oxygen sensor at the desired position over the root surface. Once positioned, it was moved on the *z* axis at 50 µm steps. Inset: example data of a recorded oxygen profile and estimation of the oxygen diffusivity. Line drawing was made from scratch using Adobe Illustrator v17.0.

Root areas prone to higher O_2_ diffusion were detected as follows. Collected intact roots were immobilized at the bottom of a methacrylate container and immersed in deoxygenated 0.15% agar solution. Roots were slightly bended in a way the base of the root was exposed to the air, while tip remained immersed in solution (Fig. 6). Roots were deepened about 3 to 3.5 cm in the agar solution. Low melting agar (1.5 g/L) was used to increase density and minimize oxygen diffusion from the atmosphere, and to minimize convective diffusion^64^. Agar solution was boiled for 20 min and bubbled with pure nitrogen gas while cooling^7^. In addition, 5 mL of an exponentially growing baker’s yeast culture (*Saccharomyces cerevisiae*) was added. The addition of an active oxygen respiring microorganism enhanced oxygen diffusion at the root surface and minimized accumulation in solution. Consequently, orthogonal profiles of oxygen concentration from the root surface could related to the diffusion rate. Maximum duration of measurements, using the same agar solution, was three hours. After this time, new solutions were prepared and degassed.

In order to estimate diffusion at different positions along the root longitudinal axis, oxygen profiles were obtained orthogonally to the root surface at different distances from the root tip. Oxygen measurements were obtained with an oxygen microsensor (tip diameter 50 µm. Unisense, Denmark) and a micromanipulator. Potential oxygen diffusion rates (pOD, μmol O_2_/L/μm) were calculated as the tangent at the root surface of O_2_ concentration *vs* distance curves^7^.

### DNA extraction

Total DNA was extracted from 1.0 g of sediment and 0.5 to 1.0 g of roots using a combination of enzymatic cell lysis in the presence of lysozyme (final concentration 1 mg/mL, at 37 °C for 45 min), and proteinase K (final concentration 0.2 mg/mL, at 55 °C for 1 h), followed by a CTAB (cetyl trimethylammonium bromide) based extraction protocol^65^. Extracted DNA was rehydrated in 50 µL of 10 mM Tris–HCl buffer (pH 7.4). DNA concentration was determined using Qubit 2.0 (Invitrogen, Molecular Probes Inc., Oslo, Norway). DNA extracts were stored at − 20 °C.

### Determination of bacterial and archaeal abundance by qPCR

The abundances of ammonia oxidizing bacteria and archaea (AOB and AOA, respectively) were estimated by quantifying the ammonia monooxygenase (*amoA*) gene using quantitative PCR. In addition, bacterial and archaeal 16S rRNA genes were quantified and used as a proxy of total abundances. Primers and thermal cycling conditions were used as previously described^44, 66^ with minor modifications. All reactions were performed in a LightCycler 96 Real-Time PCR system using the LightCycler 480 SYBR Green I Master (Roche Life Science, Basel, Switzerland). Standard curves were obtained using serial dilutions (from 10^1^ to 10^7^ copies per reaction) of linearized plasmids containing the respective genes. Limits for quantification in qPCR reactions could be lowered to 1,000 and 500 copies per reaction for 16S rRNA genes of Bacteria and Archaea, and to 50 and 10 copies per reaction for *amoA* genes of Bacteria and Archaea, respectively. The lowest quantifiable value was used for those samples were gene copies were not detected above the quantification threshold. qPCR efficiencies ranged between 82 and 107% in all reactions. Negative controls resulted in undetectable values. Inhibition tests were performed by mixing an internal standard (10^5^ copies of PCR4-TOPO plasmid, Invitrogen) and the DNA extract at a concentration of 5 ng/µL in the same reaction. No inhibition was assumed when less than 10% variation of the expected concentration of the internal standard was obtained. When needed, DNA extracts were diluted to avoid inhibition.

### Determination of the microbial community structure

The analysis of 16S rRNA gene community was carried out from extracted DNA. Library preparation and sequencing were performed at MSU Genomics Core (Michigan, USA) using Illumina 250PE MiSeq^67^. Amplification reactions were carried out using primers F515/R806 targeting the V4 region of 16S rRNA^68^. Dual indexed, Illumina compatible ends were added to the primary PCR products by secondary PCR with primers directed at the Fluidigm CS1/CS2 oligo ends. Base calling was done by Illumina Real Time Analysis (RTA) v1.18.54 and output of RTA was demultiplexed and converted to FastQ format with Illumina Bcl2fastq v2.19.0.

Quality of raw reads was initially checked using the FastQC application (www.bioinformatics.babraham.ac.uk). Raw sequences were demultiplexed, joined paired reads, quality-filtered, chimera checked and clustered into operational taxonomic units (OTUs) (97% cut-off) using Usearch v.9.1^69^. Sequences were quality-filtered using a maximum expected error of 0.25 and a minimum sequence length of 250 bp. Singletons and doubletons, OTUs containing one or two sequences, were removed to avoid spurious diversity. Paired-end sequences were aligned and classified using Mothur v1.39^70^. Taxonomic classification of the OTU representative sequences was done using the SILVA 128 reference alignment and taxonomy database. Sequences yielding < 50% bootstrap or unclassified genera with the SILVA database were taxonomically identified by Blast (NCBI).

Alpha-diversity indicators of richness and diversity (Shannon and phylodiversity indices) were calculated in Mothur after normalization of the number of sequences in each sample by randomly selecting a subset corresponding to the lowest amount of sequences found in a sample.

Weighted Unifrac distances were calculated as a measure of changes in the community composition between samples (beta-diversity). Samples were latter clustered in a PCoA. Indicator species tests^71^ were performed using Mothur and primer-e v6^72^. Significant differences between groups according to root section and/or geographical area were tested with PERMANOVA test, using primer-e v6.

### Statistical analyses

Normal distribution and homoscedasticity of oxygen diffusion and genes quantification data were analysed by Kolmogorov–Smirnov and Levene tests. Gene abundance data were log transformed prior to any statistical test. Despite this transformation, no normal distribution of data was achieved for oxygen diffusion (Kolmogorov Smirnov test, p < 0.05), and non-parametric tests were used^36^. Wilcoxon test for paired samples was conducted to identify differences in pOD rates among root sections. Differences in log transformed gene abundance data were tested between samples grouped according to their geographical location and root section by using parametric t-tests. Correlations of gene abundances, estimated oxygen diffusion rates, and physicochemical parameters were performed using Spearman’s correlation test. The significance level for all tests was set at 0.05. All analyses were performed using SPSS 23.0 (IBM SPSS, Inc).

## Supplementary information

Supplementary file1
