# Supplementary material for: Limited effect of radial oxygen loss on ammonia oxidizers in Typha angustifolia root hairs
Source: Sci Rep. 2020 Sep 24;10:15694. doi: 10.1038/s41598-020-72653-9 (PMC7518425; doi:10.1038/s41598-020-72653-9)
Supplement: Supplementary file 1 — Supplementary file1 [file 41598_2020_72653_MOESM1_ESM.docx]

**SUPPLEMENTARY INFORMATION
Limited effect of radial oxygen loss on ammonia oxidizers in *Typha angustifolia* root hairs**

Elena Hernández-del Amo1, Iva Dolinová2, Gal·la Ramis-Jorba1,3, Frederic Gich1, Lluís Bañeras1*

**Supporting Table S1.** Number of total sequences obtained per sample and alpha diversity indices of each sample. S_obs_ = number of OTUs observed, H’ = Shannon index, PD = PhyloDiversity index. Mean and SE of triplicates are shown except when indicated.

|  | **Section** | **#filtered sequences** | **S_obs_** | **H'** | **PD** |
| --- | --- | --- | --- | --- | --- |
| **Empuriabrava Constructed Wetlands** | | |  |  |  |
| **Europa Lagoon** | **Base** | 90,300 | 2,832±482 | 6.95±0.18 | 97.69±18.35 |
|  | **Middle** | 69,898 | 2,036±1,598 | 6.51±0.62 | 82.12±41.8 |
|  | **Sediment** | 108,318 | 3,450±108 | 6.98±0.04 | 107±0.61 |
| **Cell 1** | **Base** | 105,443 | 1,639±57 | 5.24±0.16 | 63.04±6.4 |
|  | **Middle** | 135,780 | 1,452±67 | 4.86±0.15 | 53.46±11.25 |
|  | **Tip (n=1)** | 32,492 | 3,530 | 7.11 | 152.42 |
|  | **Sediment** | 140,984 | 3,123±87 | 6.86±0.2 | 104.28±9.82 |
| **Cell 2** | **Base** | 147,922 | 2,145±234 | 5.94±0.28 | 75.02±5.6 |
|  | **Middle** | 115,823 | 1,768±706 | 5.2±0.66 | 63.58±17.32 |
|  | **Tip (n=1)** | 24,457 | 773 | 5 | 42.09 |
|  | **Sediment** | 113,304 | 2,356±1,212 | 5.92±1.36 | 91.93±40.77 |
| **Baix Ter** |  |  |  |  |  |
| **Bassa Tortugues** | **Base** | 98,799 | 158±32 | 3.65±0.43 | 10.85±3.18 |
|  | **Middle** | 295,797 | 897±379 | 4.82±0.4 | 20.43±5.54 |
|  | **Tip** | 86,359 | 932±83 | 4.29±0.01 | 36.22±11.73 |
|  | **Sediment** | 368,587 | 1,180±718 | 4.69±1.19 | 30.79±17.19 |
| **Daró River Mouth** | **Base** | 271,082 | 2,440±1,004 | 6.43±0.59 | 54.75±23.45 |
|  | **Middle** | 222,485 | 2,498±812 | 6.4±0.13 | 63.96±16.56 |
|  | **Tip** | 170,788 | 2,653±216 | 6.5±0.26 | 60.24±15.18 |
|  | **Sediment** | 375,224 | 2,815±1,857 | 5.52±3.32 | 58.11±39.68 |
| **Rec Coll** | **Base** | 233,031 | 985±696 | 4.8±0.53 | 26.36±14.61 |
|  | **Middle** | 240,514 | 1,979±113 | 5.4±0.51 | 50.73±12.02 |
|  | **Tip** | 96,659 | 385±336 | 4.28±0.67 | 12.75±7.11 |
|  | **Sediment** | 292,905 | 1731±1324 | 5.72±1.78 | 40.91±36.51 |

**Supporting Table S2.** List of taxa (defined phylogenetic groups or genera) of putative ammonia oxidizing microorganisms according to genomic databases that have been found in the samples analysed in this study.

| **Genus** | **Ammonia oxidizer group** | **Reference** |
| --- | --- | --- |
| AK59 *Thaumarchaeota* | AOA | Yang et al 2016 |
| Group C3 *Bathyarchaeota* | AOA | Gubry-Rangin, et al 2014 |
| Marine Group I *Thaumarchaeota* | AOA | Pester et al 2011 |
| *Candidatus* Nitrosoarchaeum | AOA | Kim et al 2011 |
| *Candidatus* Nitrosopumilus | AOA | Mosier et al 2012 |
| Soil Crenarchaeotic Group (SCG) | AOA | Chen et al 2008 |
| *Candidatus* Nitrososphaera | AOA | Spang et al 2012 |
| *Nitrosomonas* | AOB | Purkhold et al 2000 |
| *Nitrospira* | AOB (comammox) | Daims et al 2015 |

**Supporting Figure S1.** Composition of SEM images of root surfaces. Different parts of the roots are shown from the tip (upper images), to the basal section (lower images). Cells, bacilli or cocci, are clearly visible on the root surface (examples marked white arrows)

**Supporting Figure S2.** Box plots showing the abundance (copies/gFW) of 16S rRNA and *amoA* genes according to the sampled environments and sections of the root. Abundance of genes in sediments is also shown in each of the sampled environments for comparison. Distances from the root apex: Tips- 0 to 15 mm, Middle- 15 to 45 mm, Base- > 45 mm.

**Supporting Figure S3.** Relative abundances (16S rRNA sequences) of main phyla in sediments and root samples according to root section and sampled environment. “Other” refers to phyla that represented < 1% of total sequences in all samples. Total number of sequences (N) is indicated on the right of the graph.

**Supporting Figure S4.** PCoA distribution of samples according to putative microbial community composition determined by Unifrac weighted matrix. Vectors show the correlation of environmental variables to PCoA axis 1 and 2. Sample labelling, Cell 1 and 2- Sampling points 1 and 2 and the treatment cells of the Empuriabrava FWS-CW, EE- Europa Lagoon, BT- Bassa de les Tortugues, DD- Daró river mouth, RC- Rec Coll.


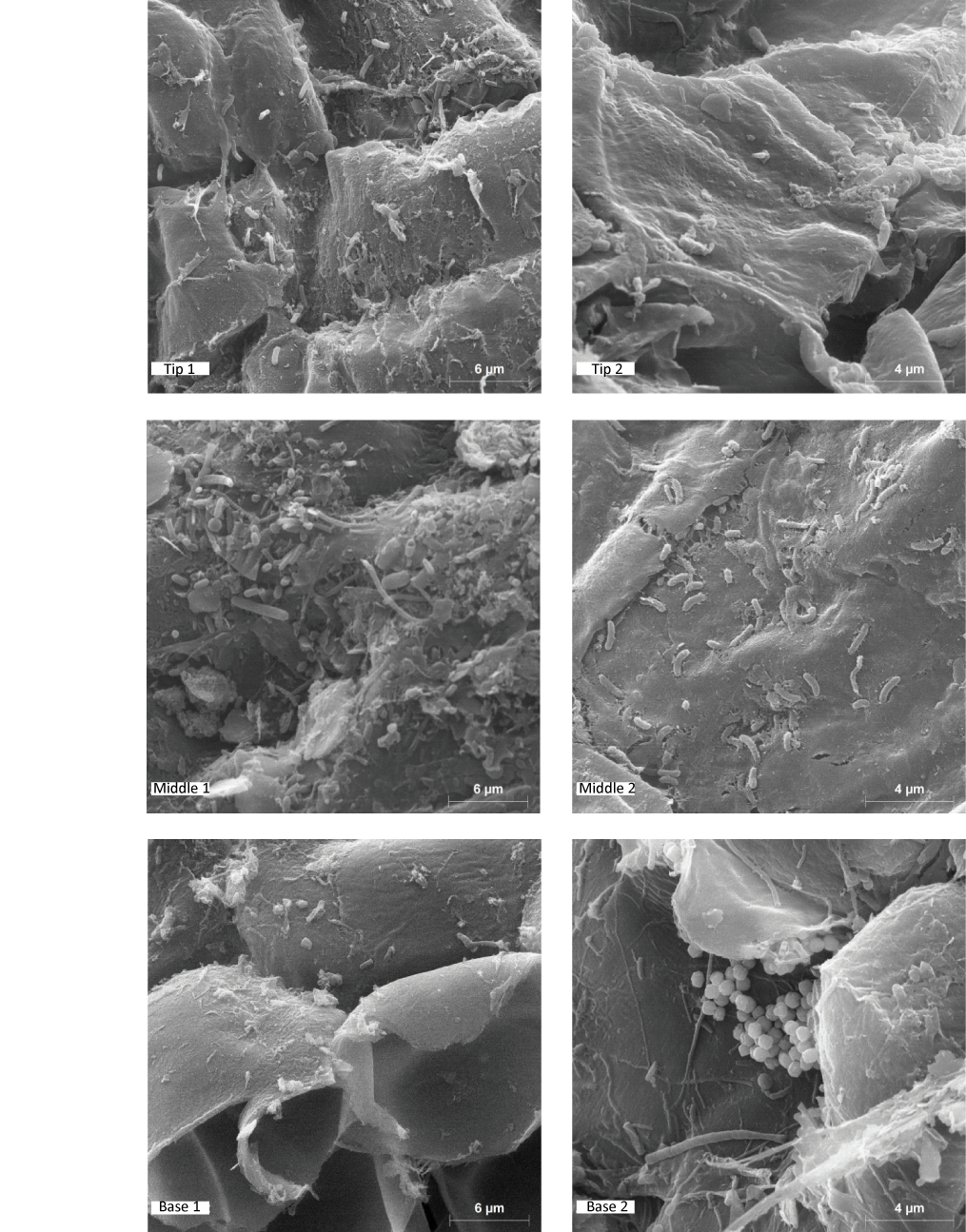


Hernández-del Amo et al **Supporting Figure S1**


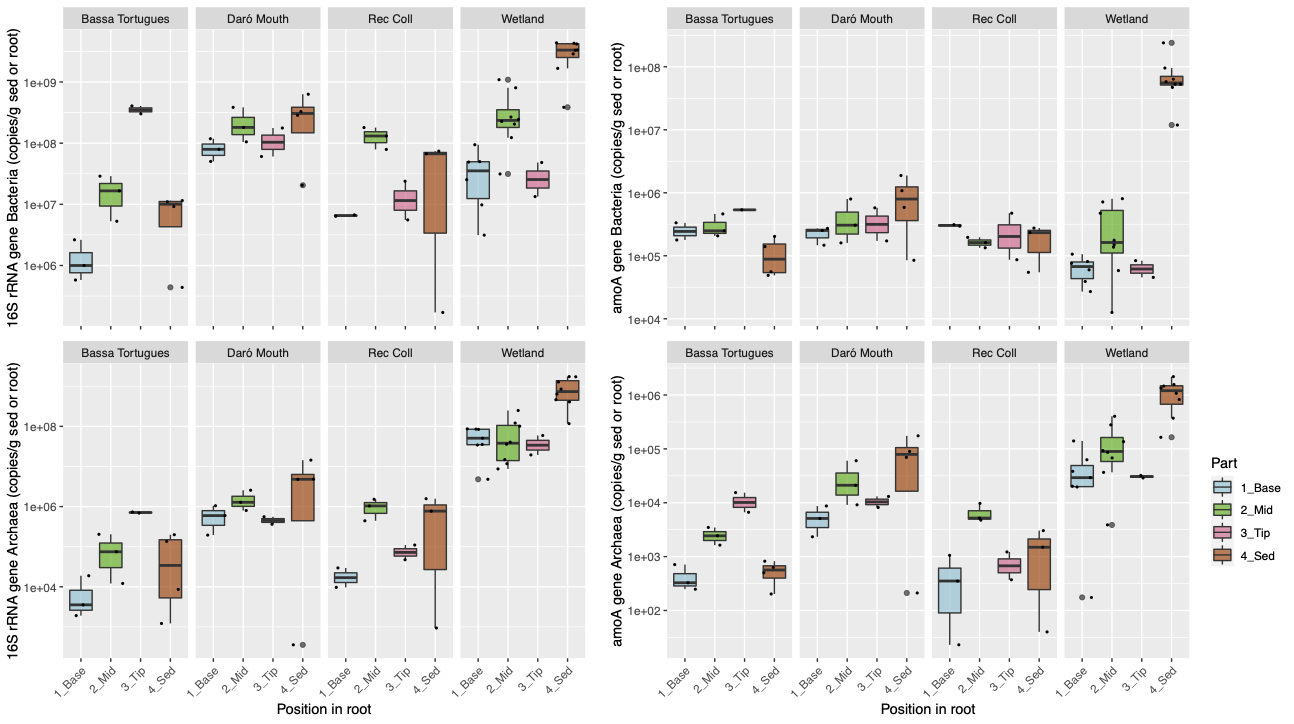


Hernández-del Amo et al **Supporting Figure S2**


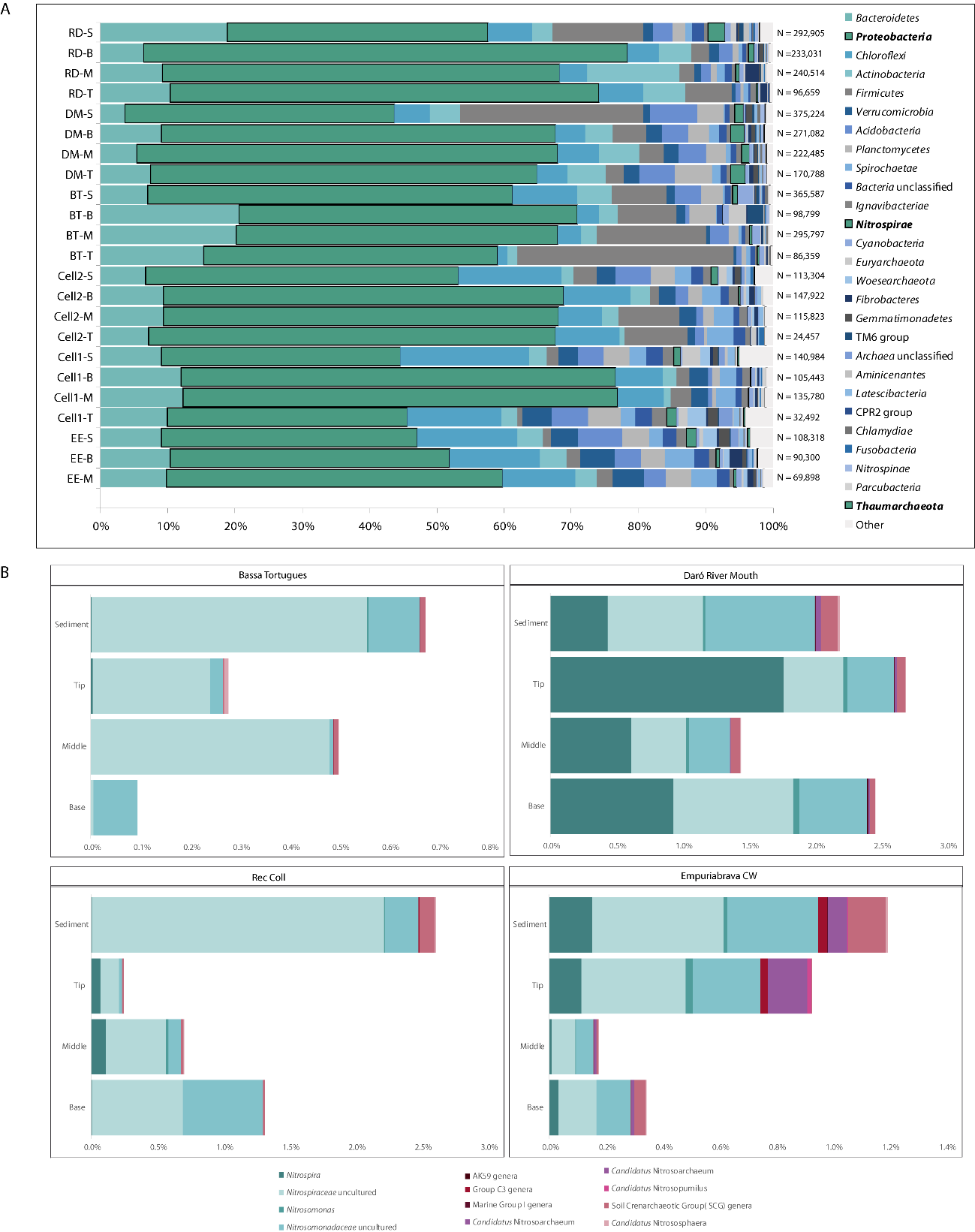


Hernández-del Amo et al **Supporting Figure S3**


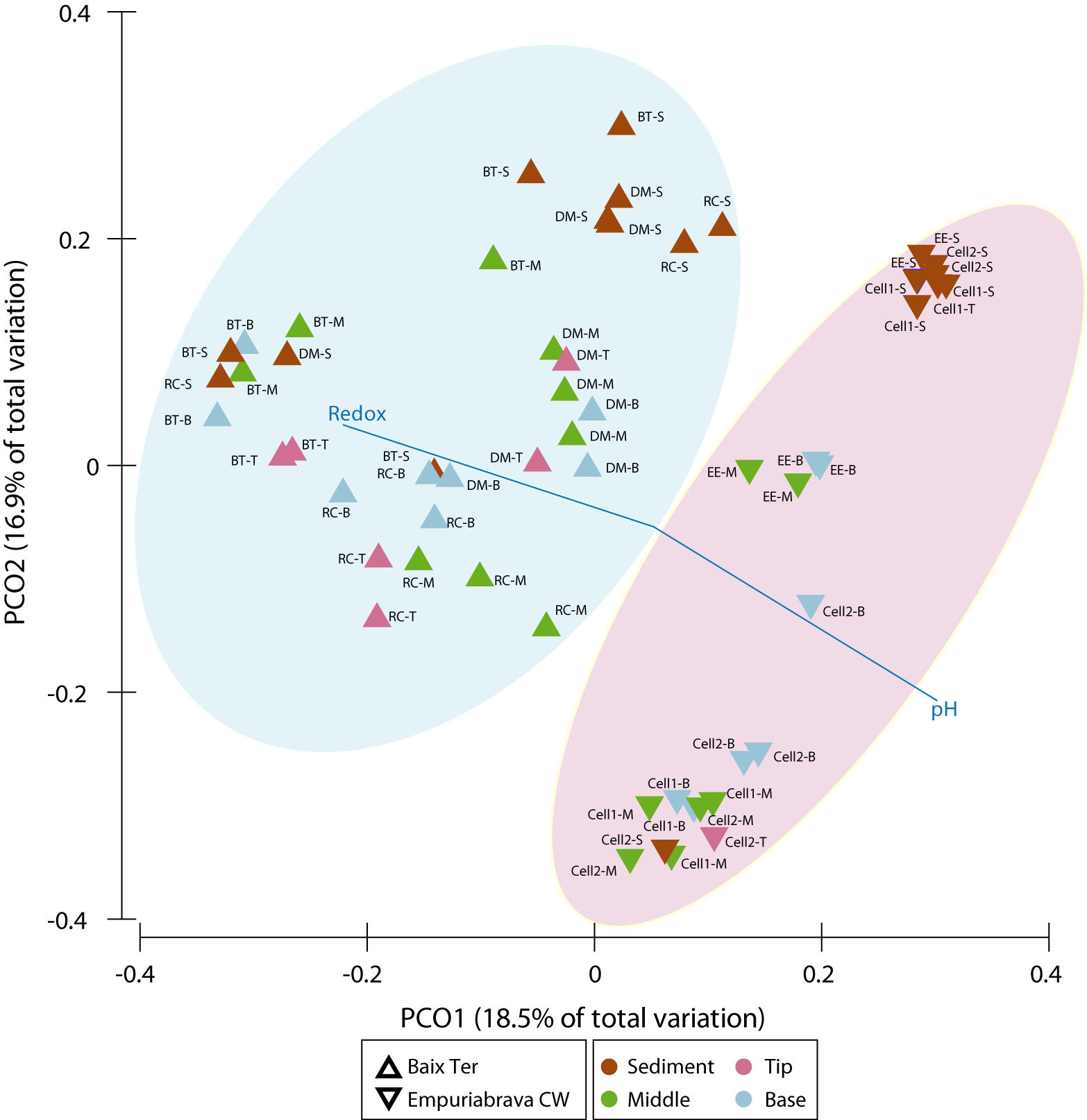


Hernández-del Amo et al **Supporting Figure S4**
